# Factors Affecting Preparation of Molecularly Imprinted Polymer and Methods on Finding Template-Monomer Interaction as the Key of Selective Properties of the Materials

**DOI:** 10.3390/molecules26185612

**Published:** 2021-09-16

**Authors:** Aliya Nur Hasanah, Nisa Safitri, Aulia Zulfa, Neli Neli, Driyanti Rahayu

**Affiliations:** 1 Department of Pharmaceutical Analysis and Medicinal Chemistry, Faculty of Pharmacy, Universitas Padjadjaran, Jl. Raya Bandung Sumedang KM 21.5, Sumedang 45363, Indonesia; nisa18005@mail.unpad.ac.id (N.S.); aulia18007@mail.unpad.ac.id (A.Z.); neli18001@mail.unpad.ac.id (N.N.); driyanti.rahayu@unpad.ac.id (D.R.); 2 Drug Development Study Center, Faculty of Pharmacy, Universitas Padjadjaran, Jl. Raya Bandung Sumedang KM 21.5, Sumedang 45363, Indonesia

**Keywords:** molecular imprinted polymer, interaction mechanism, template-monomer interaction, MIP-template interaction

## Abstract

Molecular imprinting is a technique for creating artificial recognition sites on polymer matrices that complement the template in terms of size, shape, and spatial arrangement of functional groups. The main advantage of Molecularly Imprinted Polymers (MIP) as the polymer for use with a molecular imprinting technique is that they have high selectivity and affinity for the target molecules used in the molding process. The components of a Molecularly Imprinted Polymer are template, functional monomer, cross-linker, solvent, and initiator. Many things determine the success of a Molecularly Imprinted Polymer, but the Molecularly Imprinted Polymer component and the interaction between template-monomers are the most critical factors. This review will discuss how to find the interaction between template and monomer in Molecularly Imprinted Polymer before polymerization and after polymerization and choose the suitable component for MIP development. Computer simulation, UV-Vis spectroscopy, Fourier Transform Infrared Spectroscopy (FTIR), Proton-Nuclear Magnetic Resonance (^1^H-NMR) are generally used to determine the type and strength of intermolecular interaction on pre-polymerization stage. In turn, Suspended State Saturation Transfer Difference High Resolution/Magic Angle Spinning (STD HR/MAS) NMR, Raman Spectroscopy, and Surface-Enhanced Raman Scattering (SERS) and Fluorescence Spectroscopy are used to detect chemical interaction after polymerization. Hydrogen bonding is the type of interaction that is becoming a focus to find on all methods as this interaction strongly contributes to the affinity of molecularly imprinted polymers (MIPs).

## 1. Introduction

Molecular imprinting is a technique for creating artificial recognition sites on polymer matrices that complement the template in terms of size, shape, and spatial arrangement of functional groups. This molecular imprinting technique uses target molecules in a synthetic polymer matrix by selectively binding [1,2]. Recently, molecular imprinting technology has been used to create biometric surfaces in biosensors. There are many molecular imprinting technologies, including bulk printing, surface printing, and epitope printing.

In the bulk printing method, the template molecules are printed on the entire polymer matrix, and at the end of the method, the template needs to be removed from the polymer. The polymer produced in this method is large or bulk, so grinding must be carried out on the polymer to obtain template-specific binding sites on the polymer [1]. Due to the thick morphology of the polymer in the bulk printing, it causes low access for the target molecule to bind to its specific site. Therefore, another method was developed to overcome this limitation, namely the surface printing method and the epitope printing method.

In surface printing, the removal of template molecules will result in specific binding sites on the polymer surface [3]. The binding site on the polymer surface causes this type of polymer to provide greater access to the binding target molecule than bulk imprinting [1]. This technique has been widely used for various types of analytes such as proteins [4], cells [5], and micro-organisms [6]. While in epitope printing, the target molecule is a protein and uses only a small portion or fragment of the macromolecule is printed to represent the whole molecule (epitope) as a template [1]. In this method, the peptide epitope is covalently bonded to the silicon surface where the monomer is polymerized. In epitope printing, more specific and strong interactions can be obtained. The polymer has the ability to recognize templates as well as whole proteins very well [7].

Molecularly Imprinted Polymer (MIP), which contains specific bonds between template molecules and polymers [8], is an example of materials that use molecular imprinting techniques. MIP is a unique recognition system resulting from templates and functional monomers that are polymerized, enabling molecular recognition utilizing principles similar to those underlying the action of enzymes and their substrates [9,10]. The main advantage of MIP is high selectivity and affinity for the target molecules used in the molding process. Compared to biological systems such as proteins and nucleic acids, imprinted polymers have higher physical strength, high temperature, pressure resistance, and inertia to acids, alkalis, metal ions, and organic solvents. In addition, the synthesis cost is low, the storage life of the polymer can be very high, and the recognition capability can be maintained for several years at room temperature.

**Figure 1:**
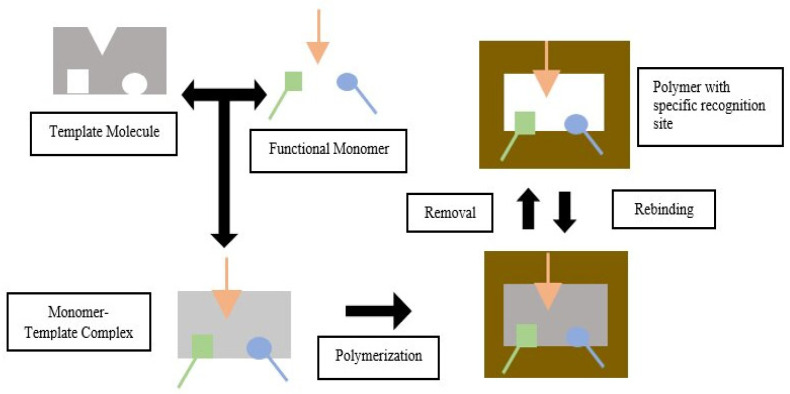
Molecular Imprinting Process (green, orange and blue arrow indicated different type of functional groups that could be appeared in the functional monomer).

Templates, functional monomers, solvent, initiator, and cross-linker are MIP components [11]. MIP is based on the formation of complexes between analytes (templates) and monomers of functional compounds. In the presence of excess cross-linking agents, three-dimensional polymers are formed [12]. In the process of making MIP, the selection of the constituent components will affect the performance of the resulting imprinted polymer. A functional monomer is preferred over an ordinary monomer because a functional monomer contains a Y functional group that can interact with template molecule via hydrogen bonding, dipole-dipole, and ionic interaction to produce a template-monomer complex. The complex is then fixed in the presence of a large excess of a cross-linking agent, and a three-dimensional polymer network is formed. After the polymerization process, template molecules are removed from the polymer using a solvent, resulting in selective complementary polymer-template bonds [11,13]. The scheme of the molecular imprinting process can be seen in Figure 1.

Several interactions occur in the molecular imprinting process, like hydrogen bonds, dipole-dipole, and ionic interactions. The interactions between the template molecule and functional groups present in the polymer matrix drive the molecular recognition phenomena. Thus, the resultant polymer recognizes and binds selectively to the template molecules. As interaction is the driving force for molecular recognition, finding the chemical interaction that happened while developing MIP is crucial to be known. According to a literature search, no review has been made on methods to find chemical interaction between template-functional monomers before and after polymerization. For that reason, this review will discuss the factors that influence the success of making MIP, especially on choosing the suitable component to form MIP and methods to determine the type of interactions happening in MIP. We will divide the method on finding the type of interaction in MIP into two stages: pre-polymerization and post-polymerization [14,15,16,17]. Thus, we will discuss computer simulation, UV-Vis Spectroscopy, Fourier transform infrared spectroscopy (FTIR), Proton Nuclear Magnetic Resonance (^1^H-NMR) for methods on finding the type of interaction in the pre-polymerization stage. In contrast, as a method for detecting the type of interaction after polymerization, we will discuss Suspended State Saturation Transfer Difference High Resolution/Magic Angle Spinning (STD HR/MAS) NMR, Raman Spectroscopy, Surface-Enhanced Raman scattering (SERS), and Fluorescence Spectroscopy.

## 2. MIP Application

### 2.1. Environmental Monitoring

The increasing contaminants in the environmental water is an alarming issue [18]. Environmental monitoring can be carried out on environmentally hazardous materials such as dyes [19], persistent organic pollutants [20], pesticide residues [21], mycotoxins [22], heavy metals, and antibiotics [23]. The presence of these contaminants can cause harmful effects on public health, either directly or indirectly [24]. Environmental matrices that can be polluted by pollutants include air, soil, atmosphere, sediment, and flora and fauna [25,26]. The pre-treatment sample aims to eliminate matrix interference so that only the target analyte is obtained [27]. MIP is commonly used as a sorbent in pre-treatment samples in samples with complex matrices because of its selected properties compared to conventional sorbents [26].

One of the applications was presented by Song et al. [28] that analyzed ten macrolide drugs (spiramycin, clarithromycin, erythromycin, tulathromycin, midecamycin, roxithromycin, josamycin, kitasamycin, tilmicosin, and azithromycin) in environmental water. MIP with functional monomer methacrylic acid and tylosin as a template was used as a sorbent in the molecular imprinted solid-phase extraction (MI-SPE) pre-treatment technique and then analyzed using the LC-MS/MS method. As a result, MI-SPE showed a high recognition ability of macrolides. The mean percentage recovery of macrolides at four spiked concentration levels was 62.6–100.9%, with intra-day and inter-day relative standard deviations below 12.6%.

### 2.2. Food Analysis 

Contaminants from the environment can enter the body through food or drinking water and have the potential to cause harmful effects on health [29]. For example, insecticide and herbicide contaminants used in agriculture include fruits, vegetables, and cereals. Effective analytical methods and technologies are needed to ensure food safety. Food is a complex matrix sample, so MIP can be used as a sorbent in the analysis process [30].

Garcia et al. [31] assessed dimethoate spiked in olive oil using core-shell magnetic-photonic dual responsive molecularly imprinted polymers (magnetic-photonic DR-MIP) as sorbent. Sample preparation using a DR-MIP-based sorbent is superior to the MI-SPE technique in terms of the procedure and minimized processing time. As a result, DR-MIP was declared a promising sorbent for the analysis of spiked dimethoate olive oil in sample preparation because it produced a high percentage recovery of 83.5% ± 0.3% with a low detection limit of 0.03 μg/mL [31].

Besides olive oil, MIP is also used for the analysis of macrolide antibiotics in honey, milk, and drinking water samples [32], porcine serum albumin in raw meat extract [33], lincomycin residue in pasteurized milk [34], triazine pesticides on cereals [35], fluoroquinolones in fish samples [36], and so on.

### 2.3. Biomedical Diagnostic

In biomedical applications, MIP is known as an artificial receptor that has basic capabilities like natural receptors, one of which is the ability to recognize cells [37]. Compared to natural receptors, MIP has advantages such as high affinity and selectivity, more temperature stability, rapid preparation, and low costs [38]. MIPs are designed to have specific recognition sites for target molecules such as antibodies and enzymes [38,39]. In the immune system, antibodies must be specific in recognizing certain antigens. MIP as an alternative to antibodies is often used as an analytical method for diagnostic purposes [40], such as breast cancer diagnostics [41], cardiovascular disease [42], and dengue fever [43]. Therefore, MIP acts as a biomarker [44] because it is able to show certain physical characteristics or measurable biologically generated changes in the body associated with certain diseases or health conditions [45].

### 2.4. Drug Delivery

Drug-delivery systems (DDS) are a method of delivering drugs to the desired place before drug release and absorption so as to increase the pharmacological activity of the drug and achieve the desired therapeutic effect [46,47]. DDS must be able to control the amount and speed of drug release [46]. MIP as a DDS agent offers several advantages, namely long shelf life, easy preparation, high chemical, and physical stability, and low cost [39].

Suksuwan et al. [48] evaluated the ability of drug delivery to cancer cells using an enantioselective receptor for (R)-thalidomide enantiomer designed in nanoparticles using methacrylic acid, a fluorescently active 2,6-bis(acrylamido)pyridine and N,N_0_-methylene-bis-acrylamide, via both a covalent approach and a physical approach. The results of his research show that MIP nanoparticles, through a physical approach, have the potential to make effective drugs to attack multidrug-resistant cells with the right temperature at the target location.

## 3. Choosing Right Component for MIP

Many factors can influence the success of making molecularly imprinted polymers. These factors include the properties of monomers, cross-linkers, and solvents [17,49,50]. The properties of monomers, cross-linker, and solvents will affect the morphology and size of the polymer formed and template-monomer interactions.

### 3.1. Functional Monomers

Functional monomers are one of the components of MIP whose properties will influence the success of making a MIP. Functional monomers are an essential factor for binding interactions in molecular imprinting technology [51]. The functional monomer will influence the binding site affinity of a MIP [52]. In the MIP pre-polymerization, the functional monomers are going to interact with the template molecules [11]. The formation of a stable template-monomer complex is critical to the success of MIP [53,54]. It is commonly accepted that stronger interaction between the template and functional monomer will result in a more stable template-monomer complex prior to polymerization, and consequently, the better imprinting efficiency of the polymer resulting will be [55,56,57]. Monomers are positioned spatially around the template, and due to cross-linking, the position of the individual repeat units that interact with template molecules will become fixed. Polymer microporous matrix possessing microcavities will be formed with a three-dimensional structure complementary to that of the template [11,58].

Research conducted by Zhong M. et al. [57] on the preparation of magnetic molecularly imprinted polymers for isolation of chelerythrine (CHE) showed that stable complex between CHE as a template (T) and MAA as a functional monomer (FM) in a 1:4 ratio gave the lowest binding energy in computational calculation compared to other ratios (1:1; 1:2; 1:3; and 1:5). The lowest binding energy indicated the highest complex stability. When the imprinting ratio (template:monomer) was 1:5, the excess monomers would increase their own association. At the same time, the spatial structure of the complex was unstable due to the space steric hindrance between the template and the functional monomer molecules. The result of the wet laboratory experiment also showed that the ratio (T:FM = 1:4) gave higher selectivity and adsorption capacity for template molecules toward analogous compounds.

**Figure 2:**
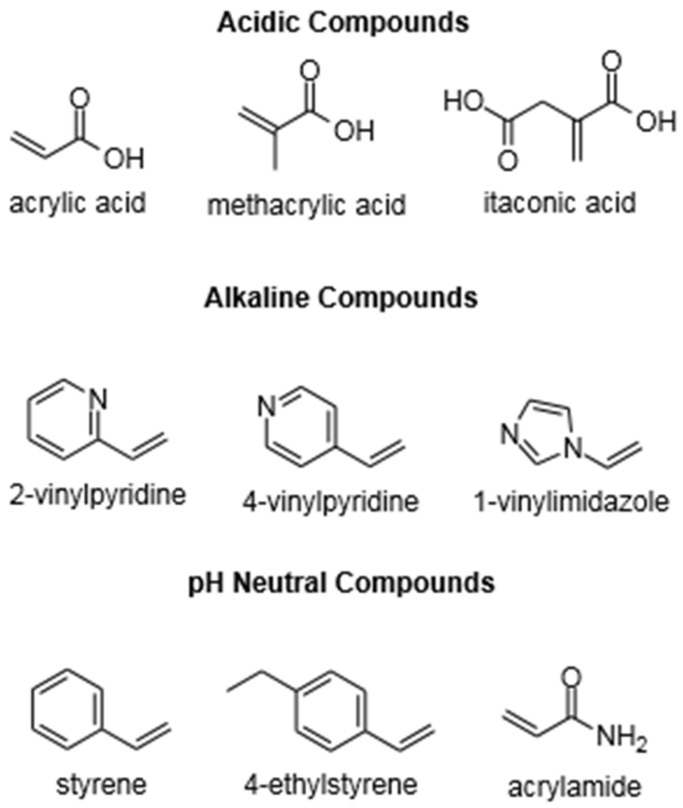
Chemical Structure of Functional Monomers.

Three types of functional monomers are most commonly used: (i) acidic compounds, such as methacrylic acid, (ii) alkaline compounds, such as 4-vinyl pyridine, and (iii) pH neutral compounds, such as styrene [59]. Some typical functional monomers are listed in Figure 2. 

A sufficiently high number of functional monomers is required to ensure a high binding capacity for the target molecule [60]. If the amount of monomer is too large, it usually results in a more non-specific interaction site [50]. The use of correct proportions of monomers and templates in the pre-polymerization process will lead to the formation of many high-affinity sites [60]. The effect of monomer amount on MIP was studied by Zhao et al. [61]. They studied the effect of amount variation of methyl methacrylate (MMA) as a functional monomer on the adsorption performance of a Solasenol MIP (SSO-MIP). Their research proved that the adsorption performance of a MIP would reach its highest point at a certain monomer concentration, and increasing the concentration after reaching the optimum point caused a decreased adsorption capacity value (Q). The highest point of SSO-MIP adsorption capacity, 43 mg/g, occurred when the MMA concentration was 0.2 mmol. When the MMA concentration was increased to 0.25 mmol, the adsorption capacity decreased to 41 mg/g, as well as at concentrations of 0.30 mmol (Q = 37 mg/g) and 0.35 mmol (Q = 36 mg/g) [61].

The interaction between template and functional monomer must be interdependent, i.e., based on the nature of the template/analyte and its functional monomer (acid analyte: alkaline functional monomers; alkaline analyte: functional acid monomer) [62], like the study of Xu et al. [63] who synthesized MIP Hexamethylenetetramine. Hexamethylenetetramine is an alkaline compound, and they used methacrylic acid as the monomer. Meanwhile, in another study by Zunngu et al. [64], they succeeded in synthesizing MIP with ketoprofen (acid) as a template and using 2-vinyl pyridine (alkaline) as a monomer.

The choice of functional monomer in MIP synthesis should also be based on the interactions formed by the template based on the functional groups in the selected template. Like the study of Barros et al. [50], who synthesized MIP using hydrochlorothiazide as a template. Hydrochlorothiazide has three interaction sites, one site on the thiazide ring and two sites on the sulfonamide group. These groups can form hydrogen bonds with carboxyl, hydroxyl, amine, and even amide groups. Therefore, various monomers (methacrylic acid, allylamine, 4-vinyl pyridine, methacrylamide, acrylamide, acrylic acid, and 2-(trifluoromethyl)acrylic acid) were investigated, which may interact through hydrogen bonding with hydrochlorothiazide. The choice of monomer is based on the interaction energy formed between the monomer and hydrochlorothiazide through computational simulations. It is known that methacrylic acid is the best monomer for synthesizing MIP hydrochlorothiazide because it has the highest interaction energy compared to other monomers [50].

### 3.2. Cross-Linker

The cross-linker, as a reagent of MIP, has a significant role in the formation of MIP. The role of the cross-linker is to participate in the formation of the physical characteristics of polymers in MIP. The physical characteristics of the polymer affected by the presence of the cross-linker are the morphological characteristics of the absorbent structure, three-dimensional structure, optimal stiffness, and the durability of the MIP [59,65].

There are several important factors in the use of cross-linkers, including the type and number of cross-linkers used for polymerization. The higher number of cross-linkers makes it possible to obtain a stable porous material [17]. A study was carried out by Zhao et al. [61] to see the effect of cross-linker amount on adsorption performances of a MIP. Their research proved that the adsorption performance of a MIP would increase to a certain concentration of cross-linker. After reaching optimum conditions, the further addition of the cross-linker will not increase the adsorption performance; it will even decrease the adsorption performance. The chemical compounds most often used as cross-linkers are trimethylolpropane trimethacrylate (TRIM), divinylbenzene (DVB), and ethylene glycol dimethacrylate (EGDMA) [59,66].

The presence of a cross-linker also affects the morphology of MIP that will affect the binding capacity of MIP. Studies conducted by Holland et al. [67] and Rosengren et al. [68] showed similar results that lower cross-linker concentrations provide greater binding capacity. This phenomenon occurs because the mean pore diameter decreased with increasing EGDMA, which may have resulted in a greater sieving ability of these polymers, which leads to lower overall capacity. Several studies have shown a comparison of the cross-linkers commonly used in MIP. Esfandyari-Manesh et al. [66] compared the use of TRIM and EGDMA in MIP with methacrylic acid (MAA) as functional monomer and carbamazepine (CBZ) as the template. Their study showed that MIP using TRIM (MAA-TRIM) as a cross-linker showed better binding capacity and had more carbamazepine recognition sites compared to MAA-EGDMA, with MAA-TRIM binding capacity > 300 (around 300–500) micrograms CBZ while MAA-EGDMA binding capacity was around 300 micrograms CBZ, both measured at 180 min. Another study from Pangkamta et al. [69] also, MIP with TRIM as a cross-linker showed a higher binding capacity percentage of 45–50% than MIP with EGDMA as cross-linker, which only shows 40–45%. The structure of TRIM and EGDMA possibly influences this. In the TRIM molecule, three-branched chains contain three vinyl groups, making TRIM more preferable in the polymerization process and resulting in more rigid complementary recognition sites for the template than the EGDMA molecule only has two vinyl groups. The contribution of the cross-linker, of course, also depends on other components, such as using the suitable functional monomer for the template or selecting the right solvent in the polymerization process [66,69].

### 3.3. Solvents 

Solvents act as a medium for reactions and have a significant effect on template-monomer interactions, which are certain components that must be considered to make a MIP. Solvents in the making of MIP must interact and dissolve all the starting materials but should not interfere too much during the polymerization reaction process [60]. When a high solvation value is used in the synthesis process of a MIP, the solvent will protect the molecular interaction site and weaken the strong interaction between the monomer and template, thus making the molecular recognition ability of the MIP relatively poor [14]. A study by Dong et al. [70] examined the effect of solvents on the adsorption selectivity of MIP with theophylline (THO) as a template and MAA as a functional monomer. They compared three solvents, namely chloroform, tetrahydrofuran (THF), and dimethyl sulfoxide (DMSO), and found that DMSO had the highest affinity for THO and MAA but had the lowest imprinting factor (IF), 1.0533, compared to that of THF (IF = 1.1076) and chloroform (IF = 3.3197). The Imprinting Factor (IF) value shows a particular analyte’s distribution ratio on the imprinted polymer as well as under the same conditions as the non-imprinted polymer (NIP). An IF value larger than one indicates good imprinting [71]. MIP with chloroform as solvent has the highest imprinting factor value because it provides the weakest interference on the template-monomer interaction, which results in the formation of the strongest hydrogen bonds between them [70].

MIP is usually synthesized in the organic solvent to increase the hydrogen bonding and electrostatic interactions between the template and monomer [72]. The polarity of the solvent greatly influences the template-monomer interactions. Less polar solvents promote the formation of the template-monomer functional complex, whereas more polar solvents interfere with the interactions in the template-monomer functional complex that form [17]. The statement is supported by the results of research conducted by Song et al. [73] on the effect of porogenic solvent in the manufacture of MIP for quercetin. They used four organic solvents with different polarities. The solvents are 1,4-dioxane, tetrahydrofuran (THF), acetone, and acetonitrile, from lowest to highest polarity. MIP with THF as the solvent showed a higher IF value of 1.2 compared to MIP using other solvents: 1,4-dioxane (IF = 1.05), acetone (IF = 1.07), and acetonitrile (IF = 1.03). This clearly shows that the medium polar solvent (THF) provides a better imprinting factor and the polar solvent (acetonitrile) provides the worst imprinting factor. Using a medium polar solvent (THF) will form an optimum interaction between the template and monomer and develop a uniform printing site on the MIP. When using a polar solvent (acetonitrile), the solvent interacts strongly with quercetin (template) and acrylamide (functional monomer), making it difficult for them to interact with each other. While using a less polar solvent (1,4-dioxane), a strong interaction will be formed between the template-monomer, but because the polymer’s solubility in the less polar solvent is low, the MIP formed will quickly form a precipitate [73].

## 4. Template-Monomer Interaction

A study has shown that physical properties and recognition of MIP depend on the successful interaction between template and monomer. This process occurs in the pre-polymerization stage [55]. The selected functional monomer will interact with the template, producing a stable template-monomer complex [17]. Therefore, functional monomers and templates must complement each other [59].

**Figure 3:**
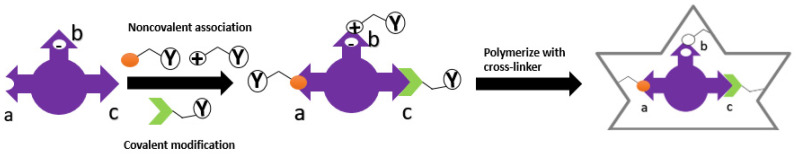
Type of interaction between template and monomer: (**a**) Noncovalent (nonionic) (**b**) noncovalent (electrostatic/ionic) (**c**) covalent.

There are currently two strategies used in MIP technology based on the nature of the template-monomer interaction. Two types of molecular imprinting strategies have been set by covalent or non-covalent interactions between the template and functional monomer [74]. An example of interaction can be seen in Figure 3. These two strategies are:

1. Self-assembly approach, which uses non-covalent bonds between monomer and template, such as hydrogen bonds, Van der Waals forces, ionic or hydrophobic interactions, and others [11]. The functional monomers are regularly positioned around the template molecules during the self-assembling process due to different exchanges [75];

2. Pre-organized approach, which uses reversible covalent bonds between the functional monomer and template. This strategy will reduce non-specific sites on MIP [11,76].

The technique most widely used in the manufacture of MIP is the self-assembly approach. In this technique, template-monomer complexes are formed in situ by non-covalent interaction [77]. Hydrogen bond, hydrophobic, and electrostatic interactions are the most widely used bonds for manufacturing MIP due to their excellent adaptability [65].

MIP manufacturing techniques using covalent and non-covalent bonds have their respective advantages and disadvantages, as listed in Table 1. 

The interaction between the template molecules and functional monomer and the formation of the polymerization mixture can lead to three different results [81]:

1. If the template is added in the pre-polymerization step, at the same time as the monomer, solvent, cross-linker, and initiator, the reaction medium will be very rich in functional monomers. The polymerization process will occur rapidly due to the dominant role of the cross-linker. A weak complex will be formed between the template and the functional monomer, producing MIP with a relatively low imprinting effect;

2. If the template is added after the start of the polymerization process, a few minutes after adding the monomer, cross-linker, initiator, and solvent, the reaction medium will contain functional macromonomers and a few monomers. Due to the nature of functional macromonomers, namely their high flexibility, they will freely re-arrange around the template to produce a MIP with a better imprinting effect;

3. If the template is added too late in the polymerization process, minutes after the optimum time of adding the template (point b), the reaction medium will be rich in pre-formed nanogel particles, which will further form a cross-linked macrogel. The condition is particularly unfavorable because the cross-linked nanogel particles are more rigid than the functional macromonomers. The MIP formed is predicted to have a low imprinting effect.

## 5. Analysis of Template-Monomer Functional Interactions

Intermolecular interactions between molecular templates and functional monomers will affect the selectivity and affinity of MIP [82]. These intermolecular interactions can be analyzed in the pre-polymerization process using computer simulation, UV-Vis spectroscopy, FTIR, and ^1^H-NMR. Meanwhile, Suspended-State STD HR/MAS NMR, Raman spectroscopy, SERS, and fluorescence spectroscopy were used after MIP formation [14,16,83,84,85,86]. Hydrogen bonding interactions are the kind of interaction that focuses on all methods as this interaction strongly contributes to the affinity of molecularly imprinted polymers (MIPs), especially for low molecular weight compounds in organic, aprotic solvents [87].

### 5.1. Computer Simulation

The development of in silico-based technology makes it easy to select MIP components such as templates, functional monomers, and suitable porogens [88,89]. In addition, it can be used to determine intermolecular interactions that occur in the pre-polymerization mixture [88]. In the pre-polymerization process, a strong template-monomer interaction will result in a suitable MIP [90]. A computational approach can be used to evaluate hydrogen bond interactions between functional template-monomer [91]. In making MIP, computational studies play a role in determining the best monomer type and ratio in a shorter time than doing experimental trials to get high selectivity [92]. Predictable functional template-monomer interactions can be found using Density Functional Theory (DFT) [83]. The DFT method can check intermolecular interactions based on the distance between the template and functional monomers and become the most extensively utilized approach in the design of MIP [70,93,94,95,96].

Wungu et al. [93] used the DFT method based on Becke three-parameter Lee-Yang-Parr (B3LYP)/6-311 + G (d) to investigate the interaction between MAA as a template molecule with D-glucose as a functional monomer. This study used Gaussian 09 software to calculate electronic properties. Before optimization, the initial structure of the MAA-D-glucose complex had a distance of 6.62 Å between the O2 atoms of MAA and the C4 atoms of D-glucose. After optimization, the distance between the MAA complexes and D-glucose was reduced to 2.81 Å, indicating non-covalent interactions with hydrogen bonds [93].

The type of interaction between the monomer templates also can be predicted using computer simulations based on the change in charge on the natural bond orbitals (NBO). NBO analysis allows for the calculation of the number of atoms in the molecule, the molecular structure, and the intermolecular or intramolecular interactions [97]. Huang and Zhu [98] conducted a computational model study to see the interaction between spermidine (template) and methacrylic acid (monomer). In theory, the two compounds can interact through hydrogen bonds. From the observation of the NBO charge, there was an interaction between the N13 atom in spermidine and the H5 atom in methacrylic acid, which was seen from the change in the NBO charge. Before complex formation, the NBO charge of N13 was −0.671, and H5 was 0.485. Meanwhile, after complex shape, the NBO charge of N13 became −0.707 and H5 became 0.501. This change in NBO charge indicates a charge transfer between the proton donor (H5) and proton acceptor (N13) in both molecules (template and monomer) and demonstrates that spermidine and methacrylic acid interact through hydrogen bonds [98].

Intermolecular interactions can also be seen through binding energy (∆E), even though we can not always determine the interaction type [93]. The initial confirmation of the respective molecular templates and functional monomers was optimized to obtain the molecule’s Gibbs free energy (∆G). The molecular templates and functional monomers are combined to produce stable complex conformations without imaginary frequencies. Functional monomers can be selected depending on the size of the ∆E [97]. The formation of the complex will be more stable as the binding energy value decreases; the lower the value of the binding energy, indicated by the negative ∆E value, the more likely the complex formed will exist in its complex form [99,100,101]. ∆E can also be used to determine the extent of the reaction, while ∆G is used to determine the spontaneity of the reaction [97]. The ∆E values were calculated using the following equation:

∆E = E (complex) − E (template) − ΣE (monomer)(1)

where E (complex) is the total energy of the template-monomer complex, E (template) is the energy of the template, and ΣE (monomer) is the energy of the functional monomer [102].

### 5.2. UV-Vis Spectroscopy 

The UV-Vis spectroscopy analytical method aims to determine the strength of the intermolecular interaction between the template and the functional monomer. The strength and affinity of the template-monomer will affect the selectivity and affinity of the polymer. Therefore, it is crucial to determine the suitable functional monomer which will interact strongly with the template to form a stable complex [52].

The interaction of template and monomer can be investigated using UV titration with nothing changes in absorbance [103]. The titration method using UV-Vis spectroscopy can evaluate the template-monomer association constant (Ka) so that the intramolecular interaction between template and functional monomer can be determined during the pre-polymerization process and also the specificity and selectivity of the polymer [104,105]. The Ka is calculated using the Benesi-Hildebrand equation [103] as follows:

(2) $$\frac{1}{\Delta Y}=\frac{1}{Y\Delta \mathrm{HGKa}\left[G\right]}+\frac{1}{Y\Delta \mathrm{HG}}$$

where ΔY is the change in absorbance, YΔHG is the change in absorbance at the end of the titration, and [G] is the concentration of the monomer added [106].

Hasanah et al. [103] used UV titration to determine the interaction between atenolol (template) and itaconic acid (functional monomer). Based on the calculation results, the association constant was 542.67 M^−1^ in methanol solvent. [103]. In another study, Hasanah et al. [107] used UV spectroscopy to determine the value of the association constant between atenolol template and Itaconic acid as a functional monomer in a mixture of methanol: acetonitrile (1:1) resulted in a constant association value of 6.277 × 10^2^ M^−1^. The interaction between atenolol and itaconic acid is a hydrogen bond from the amine groups, and the carboxylic group of atenolol and itaconic acid as the Ka value was increased in a more polar aprotic solvent [103,105]. 

Hasanah et al. [108] used UV Vis spectroscopy to see the interaction between itaconic acid monomer and diazepam as a template. The value of Ka obtained using the Benesi–Hildebrand formula was 381.9 M^−1^ ± 0.4. As stated by Wang and Yu [109], a weak interaction has a Ka value less than 25 M^−1^, and a strong interaction has a Ka value more than 100 M^−1^. So the interaction between itaconic acid and diazepam includes a strong interaction [108]. Fu et al. [52] also used UV-Vis spectroscopy to see the strength of the interaction between template luteolin and three functional monomers, namely acrylamide (AM), 4-vinyl pyridine (4-VP), and 1-aryl piperazine (1-ALPP) with different concentrations. Interaction strength was detected from significant changes of absorbance on maximum wavelength. 1-ALPP was found to cause reducing absorbance on maximum wavelength due to the π-π transition of luteolin when added in higher concentration. Meanwhile, no changes were found on AM and slight changes on 4-VP [52].

Based on the literature search, UV-Vis spectroscopy is usually used to see the strength of the interaction (from Ka value) or binding experiment. In Lulu Wang’s research in 2019 [110], a critical study was conducted to evaluate the adsorption performance of MIP and NIP for gossypol (an acidic organic compound) made with 2-(Dimethylamino)ethyl methacrylate (DMAEMA) monomer using UV spectroscopy at 373 nm by comparing absorbance values before and after absorption. More significant differences in the value represented higher binding. The gossypol molecule has a pKa value of 6.5 [110,111] and six phenolic hydroxyl functional groups -OH in its structure; it can form an acid-base ionic pair interaction with the basic amino group -NH_2_ of DMAEMA monomer. Theoretically, functional monomers containing basic groups can interact strongly with phenolic acid hydroxyl groups through acid-base interactions [112]. When we want to know the type of interaction, experiments in a few different solvents need to be run.

### 5.3. Fourier Transform Infrared Spectroscopy Analysis (FTIR)

FTIR spectroscopy can analyze samples in many forms, including liquids, solutions, gases, powders, and pastes [113]. FTIR is used in many MIP developing to identify the formation of chemical bonds, especially hydrogen bonds between molecular templates and functional monomers, due to absorption of the infrared spectrum by the sample and the peak shifting [83,114].

Xie et al. [14], in a theoretical spectrum, investigated the interaction between template chloramphenicol (CAP) and MAA functional monomer. There are peaks characteristic of strain vibrations O-H (3354 cm^−1^) and C=O (1669 cm^−1^) in the MAA spectrum. In the CAP spectrum, there are distinct peaks of O-H (3481 cm^−1^), C=O (1165 cm^−1^), and N-H stretch (3705 cm^−1^). In the spectrum of the CAP-MAA complex, which was compared with the respective MAA and CAP spectra, there was a slight shift in the stretching peaks of N-H and C=O to shorter wavenumbers, respectively 3705 to 3684 cm^−1^ and 1669 to 1657 cm^−1^. The peak shift to the lower wavenumber in the spectrum of the CAP-MAA complex compared to the pure CAP and MAA spectra indicates that hydrogen bonds are formed between CAP-MAA [14].

Analysis of the functional template-monomer intramolecular interaction of hydrogen bonds using FTIR was carried out in the study of Xie et al. [83] with deltamethrin (DM) as a template and AM as a functional monomer. In the AM spectrum, there is a peak characteristic of the NH stretching group at wavenumbers 3354 and 3184 cm^−1^. Meanwhile, In the DM spectrum, there is a peak characteristic of the C=O group at wave number 1735 cm^−1^. Compared to the single spectrum of AM, the DM-AM complex shows a shift in the peak of the NH stretching to shorter wave numbers 3346 and 3167 cm^−1^. This peak shift indicates the formation of hydrogen bonds. The hydrogen bond formation is due to the reaction between the C=O group from DM with -NH from AM [83].

Tadi and Motghare [115] also examined the hydrogen bond interaction between oxalic acid (OA) and AM as a functional monomer at the OA: AM (1:4) ratio. At a wavelength of 2640 cm^−1^, a shift occurred, which is thought to result from NH stretching of four AM monomer units. In addition, there are hydrogen bonds in the OH group (3421 cm^−1^) and hydrogen bonds in the C=O group (1650 cm^−1^), which form broad peaks [115]. Therefore, it can be concluded that the analysis of hydrogen bonding interactions between templates and functional monomers using FTIR is influenced by the electron distribution, which will cause a shift of the peak to a lower wavenumber. The formation of hydrogen bonds can be determined based on the results of MIP analysis before and after extraction [97,103,116].

### 5.4. Proton-Nuclear Magnetic Resonance (^1^H-NMR) 

^1^H-NMR can be used for the analysis of complex formation during pre-polymerization; this method is commonly used to obtain optimal MIP preparation with the appearance of non-covalent compounds (hydrogen interactions, electrostatic interactions, and hydrophobic interactions) [117,118,119]. Studies by Quaglia et al. [120] have demonstrated the use of ^1^H-NMR on the significance of hydrogen bonding in achieving imprinting effects, while work by Whitcombe et al. [121] showed how NMR investigations of monomer–template dissociation constants can predict MIP binding capacities. 

During the formation of hydrogen bonds, there is a downfield shift caused by the reduced electron density around the hydrogen nucleus [83]. The reduction in ^1^H-shielding correlates with the shortening of the distance between the donor and acceptor of the hydrogen bond. The ^1^H-shielding value of hydrogen bonding protons is determined by the reduced electron charge density around the hydrogen atom. The greater the deshielding proton, the shorter the hydrogen bond distance [119]. The downfield shift of the NMR signal of the bridging proton in a hydrogen bond is divided into two phenomena. First, the formation of hydrogen bonds due to polarization and charge transfer leading to deshielding. Second is the presence of proton-accepting groups, where the response to an external magnetic field and electron density causes an effect on the position of the proton bridge, exclusive of any H-bonding phenomenon [122]. The interaction between template molecules and functional monomers will be stronger along with the significant chemical shift [83]

The research of Xie et al. [83] reveals the hydrogen bond interaction between and AM from the chemical shifts in the single AM spectra compared to the DM-AM complex spectra. In the AM spectra, the peak of the amino hydrogen atom, which was at 3.10 ppm, was shifted to 2.89 ppm in the DM-AM complex spectra. The hydrogen bond of the carbonyl group in DM with the amino group in AM causes the electron density around the AM amino hydrogen atom to decrease so that a downfield shift occurs. This indicates that the DM template and AM functional monomer are successfully conjugated [83,123]

Sánchez-González et al. [55] investigated the interaction between template cocaine hydrochloride (COCH) and functional monomers methacrylic acid (MAA) and ethylene dimethacrylate (EDMA). The investigation was carried out by varying the molar ratio in the range 0.3–3.0 between COCH: MAA and COCH: EDMA, then comparing the chemical shift disturbance caused by certain functional groups present in the template and functional monomers. The result is a chemical shift at 2.84 ppm COCH (H from the protonated COCH), 1.89 ppm MAA (H close to the acid group in MAA), and 4.36 ppm EDMA (H close to the ester groups in EDMA). This shift occurs due to the interaction of the amino group hydrogen bonds in COCH with oxygen in the acid group (MAA) and oxygen in the ester group (EDMA) [55].

Wang et al. [99] also investigated the intermolecular interactions between the oblongifolin C (OC) template derived from the fruit extract of Garcinia yunnanensis Hu and the functional monomer AM. The molar ratio used is OC:AM = 1:4. When the single spectrum of OC was compared to the mixed spectrum of OC:AM, the proton -OH peak at 9.912 ppm experienced a downfield shift to 9.916 ppm. When the single spectrum of AM was compared to the mixed spectrum of OC:AM, the -NH_2_ proton peak at 7.509 ppm underwent a downfield shift to 7.513 ppm. When the spectrum of the ^13^C-NMR mixture (OC:AM = 1:4) was compared with the AM spectrum, the -C=O group at 166.33 ppm experienced a downfield shift to 166.35 ppm. The results of this chemical shift confirm the occurrence of intermolecular interactions between OC and AM in the form of hydrogen bonds [99]. 

As for MIP, which is formed non-covalently with electrostatic (ionic) interactions, for example, 2,4-D herbicide, with 4-vinyl pyridine as a functional monomer, and synthesized using methanol/water solvent so that the nature of the interaction that will form is impossible for hydrogen bonds to form but possible for the electrostatic interaction. An NMR titration study of the system revealed that the primary interaction is based on the electrostatic effect. The basic pyridine molecule and the 2,4-D acid molecule form an ionic pair, and a solvent is also used. Controls use a non-polar solvent (deuterated chloroform); this solvent is used because observing interactions in the solvent (methanol) is complicated due to the exchange process between the labile protons of the analyte and the deuterium atom of the solvent takes place rapidly [124]. The acidic proton signal of the carboxylic acid migrates strongly upwards as the titration progresses for a higher concentration of 4-VP, as the 4-VP in solution is progressively protonated. The electrostatic interactions supported by the imprinted polymer show the most excellent affinity for the structural analogs of phenoxy acetic acid and 2,4-dichlorobenzoic acid. Both compounds can interact electrostatically with their carboxylic substituents, and both contain an aromatic group so that the affinity for 2,4-dichlorobenzoic acid shows steric [125].

### 5.5. Suspended-State High Resolution/Magic Angle Spinning Nuclear Magnetic Resonance Spectroscopy (STD HR/MAS NMR)

Analysis using Suspended-State STD HR/MAS NMR involves solids (MIP) suspended or dispersed in a liquid. Suspended state NMR can be used to investigate the interaction between templates/analytes and MIP [85]. In this technique, the protons in the MIP are saturated. Energy will be transferred from the MIP protons to the protons in the analyte, and an increased signal in the analyte protons will occur. This increase in the signal can provide information about the possible interactions of MIP with the analyte [126].

Like the study of Courtois et al., who researched the interaction of MIP and template (bupivacaine) using suspended-state STD HR/MAS NMR. The MIP-bupivacaine interaction was observed from the increase in proton signaling of bupivacaine. Interaction observations were carried out on monolithic MIP (mMIP), and monolithic non imprinted polymer (mNIP). The mMIP showed significantly stronger interactions with aromatic protons and twice as many with nitrogen-bound protons than mNIP, thus indicating that mMIP-bupivacaine interacts via a polar interaction. While mNIP showed a signal twice as strong as the methyl group on the alkyl chain (non-polar interaction). The analysis results using STD NMR were following the chromatography results, namely bupivacaine in mMIP formed a specific binding site with the polar group of the functional monomer (MAA) [85].

Another study was from Skogsberg et al. [127], who used STD HR/MAS NMR to investigate the interaction of 9-ethyladenine (9EA) as a template/analyte and MIP. From the STD HR/MAS NMR spectrum, it was known that the signal increase occurs in several hydrogen atoms (H-2, H-8, H-10, and H-12), indicating that H-12 has the most prominent signal increase. From the observations, it was also known that the primary interaction of EA and MIP occurs through hydrogen bonds, namely through the amino group in 9EA. From the spectrum, there may be multiple hydrogen bonds between 9EA and MIP, including N-1 and N-3 (affecting H-2); N-7 (affecting H-8) [127]. Therefore, STD HR/MAS NMR is useful to see the type of interaction, whether polar or non-polar interaction between MIP and its template. 

### 5.6. Raman Spectroscopy

Raman spectroscopy can be used to detect and characterize the binding of template molecules to MIP by seeing the shifting of the Raman spectrum [84,128]. Raman spectroscopy has been extensively used to study polymerization processes and characterize polymers [129]. Raman spectroscopy is one of the promising techniques for understanding hydrogen bonds [130]. With Raman spectroscopy, laser photons will be scattered by the sample molecules resulting in a loss of energy during the process. The amount of energy lost will be detected as a change in the energy (wavelength) of the irradiating photon and represent certain bonds in molecules [128].

In the study of Xi et al. [131], investigations of intra and intermolecular interactions using Raman spectroscopy were carried out on non-covalent MIPs with three minor analgesics (aspirin, caffeine, and acetaminophen) as templates using MAA as a monomer. The investigation was carried out through changes in vibrational mode frequency shifts induced at engineered binding cavities [131]. Vibrational frequencies are sensitive to small chemical environmental changes such as temperature [132], phase transitions [133], and pressure [134]. Collective Intermolecular interactions between analgesic and MIP were assumed to influence the vibrational band higher than non-specific interactions that happened in the surfaces of the polymer particles. Xi et al. [131] measured Raman spectra of analgesics and polymer in solution, with non imprinted and MIP-bound, then examined the vibrational bands sensitive to intermolecular interactions, such as C−N−CH_3_ deformation phenyl bending and CH_3_ rocking. Higher shifting of the vibrational frequencies is related to stronger hydrogen bonding. When non-specific binding exists, vibrational frequencies will shift minorly or not shift at all [131,135].

### 5.7. Surface-Enhanced Raman Scattering (SERS)

SERS is a surface-sensitive technique that enhances Raman scattering during the binding process by incorporating the incident laser and local surface plasmons on the polymer structure [86]. SERS has a vibrational spectrum that becomes characteristic of the adsorbed substance and is used to identify compounds by comparison with a reference spectrum [136]. SERS has increased the scattering signal several times from Raman by using a rough metal surface when the molecule is adsorbed because Raman has a scattering signal that is too weak [137].

Activating SERS on the target molecule in MIP with the active metal surface placed at the recognition site is essential for enhancing the adhesion of molecularly imprinted xerogels to the SERS substrate [138]. In this study, when analyte molecules selectively bound to the recognition cavity, they spontaneously adsorb onto the surface of the surrounding silver nanoparticles. Adsorption on the surface of silver nanoparticles will produce an enhanced Raman signal from the target molecule; therefore, re-binding can be seen by measuring the intensity of SERS. For example, in Liu’s study, THO (Theophylline) -MIP polymers without silver nanoparticles lacked theophylline Raman bands even though they contained as much theophylline as Ag-THO-MIPs. This proves that the silver nanoparticles embedded around the recognition cavity will increase the Raman signal from the template theophylline molecule, resulting in a clear return of the SERS band, indicating the presence of hydrogen bonds that cause the theophylline molecule to re-bind to Ag-THO-MIPs. Because of this, SERS are widely used in identifying extraction and re-binding of template molecules due to sensitivity and specificity [137].

### 5.8. Fluorescence Spectroscopy

Fluorescence methods are already known can be applied for the characterization of polymeric materials. As an example, the fluorescence of the dansyl group known can be used to investigate the polymer structure due to sensitivity to media polarity changes [139,140]. A polymer that already formed and still contained the template was suspended in dansyl glycine solution in water then fluorescence spectrofluorometer measurements were used to measure the fluorescence signal. A study by Piletsky et al. [141] on the development of MIP for D- and L-phenylalanine used a fluorescence spectrofluorometer to see the hydrophobic and electrostatic interaction that happened between the functional group of the polymer formed with the template. The size of differences in the emission wavelength spectra of dansyl glycine in water alone and after polymer being suspended can be used to indicate hydrophobic interaction and polar interaction. More enormous differences in the emission wavelength mean hydrophobic and electrostatic interactions appeared instead of polar interaction [141]. 

Another study by Takeuchi et al. [142] used methacrylic acid (MAA) and 2-(trifluoromethyl) acrylic acid (TFMAA) for the synthesis of MIPs of cinchona alkaloids. The emission maximum of free cinchonidine in chloroform/acetonitrile solution is 360 nm and was shifted to 390 nm upon binding to the TFMAA-based polymers, suggested hydrogen bond interaction between TFMAA and cinchonidine molecules [142].

### 5.9. Method Comparison for Detecting Interactions Template-Monomer and MIP-Template

Here, we summarize the advantages and disadvantages of each method for detecting interactions between template-monomer and MIP-template listed in Table 2.

## 6. Conclusions

The molecular imprinting technique uses target molecules in a synthetic polymer matrix by performing selective binding. Functional monomer and cross-linker selection in type and ratio, type of solvents, will determine the chemical interaction in pre-polymerization solution and after the formation of the imprinted polymer. This type of interaction can be predicted and identify using computer simulations, UV-Vis spectroscopy, FTIR, ^1^H-NMR for a pre-polymerization solution. While suspended-state STD HR/MAS NMR, Raman spectroscopy, SERS, and fluorescence spectroscopy were used after the polymer being formed, ^1^H-NMR is more valuable and sensitive to find chemical interaction between template-monomers besides hydrogen bonds in pre-polymerization solution. Computer simulation, especially the DFT method, is the most extensive method used in MIP design. Computer simulation can certainly predict all types of interaction happening in MIP but still need a laboratory experiment for confirmation. After polymers are formed, the suspended-state STD HR/MAS NMR and SERS is a highly sensitive method to see hydrogen or electrostatic interaction. Even the last method needs metal to interact with the molecular chromophores. Other chemical interactions besides hydrogen bonds still need to be explored from the study as this is not the only interaction happening in MIP. 

The development of molecularly imprinted polymers (MIPs) has grown fast. MIPs have been used widely in various fields using single and multi templates, such as pharmaceuticals analysis, food, and beverage quality, environmental pollutant analysis, doping analysis, and therapeutic drug monitoring. However, there are still some problems to be explored and solved:

1. Nanostructured MIP materials often suffer from problems such as the hardenest of template removal. The condition often results in MIP nanomaterials with non-uniform recognition sites;

2. Different efficient synthesis methods to produce MIP with high binding ability and selectivity are still needed to explore to maximize commercial conversion;

3. Knowing chemical interaction that happened between MIP and template with the affordable instrument still needs to be explored shortly as an instrument available now expensive for some laboratories.

## Figures and Tables

**Table 1:** Comparison Between Covalent and Non-Covalent Imprinting Techniques.

Imprinting Type	Covalent	Non-Covalent	References
Interaction	Reversible condensation reactions (ketal, acetal, esters, boronate, Schiff’s bases)	Ionic interaction, hydrogen bonding, Van Der Waals forces	[17,78,79]
Advantage	More durable and rigid types of interactions The template-monomer complex is usually stable during the polymerization process Forming a polymer with a more homogeneous binding cavity	Flexible, fast, and straightforward binding interactions Easy template molecule removal Easy template-monomer complex preparation Resulting in a MIP with high-affinity binding, greater affinity, and selectivity to the site	[17,49,51,78,80]
Disadvantage	Strong covalent interactions result in slow binding and re-binding Difficult to remove the template	Non-covalent interactions are sensitive to disruptions	[17,79]

**Table 2:** Method Comparison for detecting interactions template-monomer and MIP-template.

Tools	Advantages	Disadvantages	References
Computer simulation	Able to determine the type and ratio of suitable monomers in less time than experiments Able to predict the occurrence of unwanted interactions such as the formation of crosslinker-monomer or crosslinker-template complexes Fast, simple, economic sensing system	The results of computer simulations must still be confirmed through experiments	[90,97,143]
UV-Vis Spectroscopy	Able to determine the strength of intermolecular interactions between templates and functional monomers	It takes a long time and needs to run an experiment in a few different solvents to see the type of chemical interaction	[52,144]
FTIR	Able to analyze liquid samples, gas solutions, powders, and pastes Able to identify bond formation from the absorption of the infrared spectrum by the sample Can also be used to see quantification of the degree of polymerization and reactivity for each type of polymerizable group on the monomers	Less selective	[113,114,145,146]
^1^H-NMR	Able to detects hydrogen bonds by observing the downfield shift that occurs due to the reduced electron density around the protons during hydrogen bond formation A good tool for studying the power and geometry of hydrogen bonds	The study of hydrogen bond interactions of small molecules by NMR is often hampered by the fast exchange of species in solution.	[83,147]
Suspended-State STD HR/MAS NMR	Simple preparation Fast Quantitative data may be obtained conveniently Able to see polar and non-polar interaction between MIP and its template	A signal from MIP/analyte may overlap with solvent	[85,127,148]
Raman Spectroscopy	Provides fingerprint information on the chemical structure of the analyte based on the shift in the Raman scattering frequency Requires little or no sample preparation In the final Raman spectrum, water is negligible	Raman scattering cross-section is tiny Only a small fraction of the photons are inelastically scattered (about 1 in 10 million) Limited by low signal-to-noise because only 1 photon out of 109 incident photons undergoes Raman scattering	[131,149,150]
SERS	Highly specific, sensitive, and accurate Fast and nondestructive method	Sample decomposition is often encountered in the measurement of SERS Sensitivity of SERS to the changes of the local environment of the molecules-MIP including H+ concentration, sensing, potential, and other factors influencing accuracy of SERS result on detection of intermolecular binding SERS mechanisms require that the molecular chromophores interact with the metal interface at short distances Molecules with poor affinity to metal often require nanoparticle functionalization or molecule derivatization	[137,151,152,153,154,155,156]
Fluorescence Spectroscopy	Highly sensitive It can be used to visualize interaction on biological systems when MIP is used as a sensor	Need fluorescence monomer or other fluorescence compounds for interaction detection Background signals usually exist caused either by strong scattering of the particles, strong absorption, or background luminescence of the polymeric system.	[140,157,158,159,160,161]

## Data Availability

Data sharing not applicable.
